# Predictive Value of the CHA_2_DS_2_-VASc Score for Mortality in Hospitalized Acute Coronary Syndrome Patients With Chronic Kidney Disease

**DOI:** 10.3389/fcvm.2022.790193

**Published:** 2022-03-16

**Authors:** Yaxin Wu, Yanxiang Gao, Qing Li, Chao Wu, Enmin Xie, Yimin Tu, Ziyu Guo, Zixiang Ye, Peizhao Li, Yike Li, Xiaozhai Yu, Jingyi Ren, Jingang Zheng

**Affiliations:** ^1^Department of Cardiology, Peking University China-Japan Friendship School of Clinical Medicine, Beijing, China; ^2^Department of Cardiology, China-Japan Friendship Hospital, Beijing, China; ^3^Department of Cardiology, Guangdong Provincial People's Hospital, Guangdong Academy of Medical Sciences, Guangzhou, China; ^4^Guangdong Cardiovascular Institute, Guangzhou, China; ^5^Graduate School, Chinese Academy of Medical Sciences and Peking Union Medical College, Beijing, China

**Keywords:** chronic kidney disease, acute coronary syndrome (ACS), CHA_2_DS_2_-VASc score, prognosis, mortality

## Abstract

**Background:**

Chronic kidney disease (CKD) patients have a high prevalence of coronary artery disease and a high risk of cardiovascular events. The present study assessed the value of the CHA_2_DS_2_-VASc score for predicting mortality among hospitalized acute coronary syndrome (ACS) patients with CKD.

**Methods:**

This was a retrospective cohort study that included CKD patients who were hospitalized for ACS from January 2015 to May 2020. The CHA_2_DS_2_-VASc score for each eligible patient was determined. Patients were stratified into two groups according to CHA_2_DS_2_-VASc score: <6 (low) and ≥6 (high). The primary endpoint was all-cause mortality.

**Results:**

A total of 313 eligible patients were included in the study, with a mean CHA_2_DS_2_-VASC score of 4.55 ± 1.68. A total of 220 and 93 patients were assigned to the low and high CHA_2_DS_2_-VASc score groups, respectively. The most common reason for hospitalization was unstable angina (39.3%), followed by non-ST-elevation myocardial infarction (35.8%) and ST-elevation myocardial infarction (24.9%). A total of 67.7% of the patients (212/313) received coronary reperfusion therapy during hospitalization. The median follow-up time was 23.0 months (interquartile range: 12–38 months). A total of 94 patients (30.0%) died during follow-up. The high score group had a higher mortality rate than the low score group (46.2 vs. 23.2%, respectively; *p* < 0.001). The cumulative incidence of all-cause death was higher in the high score group than in the low score group (Log-rank test, *p* < 0.001). Multivariate Cox regression analysis indicated that CHA_2_DS_2_-VASc scores were positively associated with all-cause mortality (hazard ratio: 2.02, 95% confidence interval: 1.26–3.27, *p* < 0.001).

**Conclusion:**

The CHA_2_DS_2_-VASc score is an independent predictive factor for all-cause mortality in CKD patients who are hospitalized with ACS. This simple and practical scoring system may be useful for the early identification of patients with a high risk of death.

## Introduction

Chronic kidney disease (CKD) is an important contributor to morbidity and mortality from non-communicable diseases and has become a considerable public health issue (1–3). Patients with CKD have a high prevalence of coronary artery disease, and many of these patients die from cardiovascular disease, especially those with acute coronary syndrome (ACS) (4, 5). The early identification of high-risk ACS patients is important for assessing prognosis and guiding treatment. Current international guidelines recommend Global Registry of Acute Coronary Events (GRACE) scores to predict the cumulative risk of death and myocardial infarction (6, 7). However, derivations of GRACE scores are based on unselected and generalizable patients, and the calculation of GRACE scores is relatively complicated (8), which may limit its application in CKD patients, especially those with end-stage renal disease. The CHA_2_DS_2_-VASc score is used to assess the combination of congestive heart failure, hypertension, diabetes, prior stroke, vascular disease, and age. It is an easily calculated scoring system that can assess the risk of stroke in patients with atrial fibrillation (9, 10). All of these risk factors have been proven to be associated with cardiovascular prognosis. Recent studies also used CHA_2_DS_2_-VASc scores to predict poor prognosis in patients with cardiovascular disease, regardless of atrial fibrillation (11–13). The risk factors that are included in this scoring system are also common in CKD patients with coronary artery disease (5, 14). The objective of the present study was to evaluate the predictive value of CHA_2_DS_2_-VASc scores in hospitalized ACS patients with CKD.

## Methods

### Study Design and Population

This was a retrospective cohort study that included CKD patients who were hospitalized for ACS from January 2015 to May 2020. We consecutively enrolled patients in the Cardiology Department, China-Japan Friendship Hospital. Cases were identified using International Classification of Diseases-Clinical Modification code 9. All enrolled patients were confirmed to have at least one major coronary artery with more than 50% stenosis, determined by coronary angiography. Data on demographics, medical history, and laboratory tests were abstracted from electronic medical records. The glomerular filtration rate was estimated according to serum creatinine and the Chronic Kidney Disease Epidemiology Collaboration (CKD-EPI). Chronic kidney disease was defined by an estimated glomerular filtration rate <60 ml/min/1.73 m^2^, including dialysis. Coronary reperfusion therapy included percutaneous transluminal coronary angioplasty (PTCA) ± stenting, PTCA alone, or coronary artery bypass grafting. The study conformed to the Declaration of Helsinki and was approved by the Research Ethical Review Committee of China-Japan Friendship Hospital (2020-112-K71).

### CHA_2_DS_2_-VASc Score

For each patient, the CHA_2_DS_2_-VASc score was calculated at admission based on the following scoring system: (1) point for congestive heart failure, hypertension, 65–74 years of age, diabetes mellitus, vascular disease, and female sex and (2) points for ≥75 years of age and prior stroke or transient ischemia attack. We performed a receiver operating characteristic analysis that showed that the best cut-off value of the CHA_2_DS_2_-VASc score to predict mortality was ≥6 with 45.7% sensitivity and 77.2% specificity [area under curve: 0.64; 95% confidence interval (CI): 0.58–0.71, *p* < 0.001; Supplementary Material]. Therefore, the CHA_2_DS_2_-VASc score was classified as <6 and ≥6. The patients were not further divided into more than these two groups because of the relatively small sample size.

### Follow-Up and Outcome

The primary outcome of the study was all-cause mortality, which was the rate of death from any cause from the date of admission until the occurrence of endpoint events or until the latest follow-up date (June 1–July 1, 2021). Clinical events were ascertained by longitudinally tracking patients' medical records or through telephone interviews.

### Statistical Analysis

Continuous variables are expressed as the mean ± standard deviation or median and interquartile range and compared using *t*-tests or the Mann-Whitney *U-*test when appropriate. Categorical variables are expressed as frequencies and percentages and were compared using the χ^2^-test or Fisher's exact test. Univariate and multivariate Cox regression analyses were performed to determine risk factors for all-cause death, and the hazard ratio (HR) and 95% CI were calculated. Variables with values of *p* < 0.10 in the univariate analysis were included in the multivariate analysis. Time-dependent survival between groups was evaluated using Kaplan-Meier curves and the Log-rank test. Stratified analyses were performed using the following variables: age (≥65 vs. <65 years), sex, hyperlipidemia, diabetes, prior myocardial infarction, hemodialysis, main diagnosis, left ventricular ejection fraction (≥50 vs. <50%), and reperfusion therapy. Multiplicative interactions were calculated in each subgroup. All statistical analyses were performed using SPSS 27.0 software (IBM Corp., Armonk, NY, USA). Two-tailed values of *p* < 0.05 were considered statistically significant.

## Results

A total of 313 eligible patients were recruited in the study. Baseline characteristics are presented in Table 1. Among these patients, the mean CHA_2_DS_2_-VASC score was 4.55 ± 1.68. A total of 220 patients (70.3%) had a low CHA_2_DS_2_-VASc score (<6 points), and 93 (29.7%) had a high CHA_2_DS_2_-VASc score (≥6 points). The high CHA_2_DS_2_-VASC score group included patients who were older and had a higher prevalence of comorbidities, including diabetes mellitus, heart failure, and cerebrovascular disease. Patients who were diagnosed with non-ST-elevation myocardial infarction (35.8%) and unstable angina pectoris (39.3%) were more common than patients who were diagnosed with ST-elevation myocardial infarction (24.9%). Among the 313 patients, 67.7% (212) received coronary reperfusion therapy, including PTCA ± stenting (*n* = 187), PTCA (*n* = 15), and coronary artery bypass grafting (*n* = 10). Accordingly, in-hospital treatment was comparable between the two groups.

**Table 1:** Baseline characteristics in CKD patients hospitalized with ACS by CHA2DS2-VASc score.

**Characteristic**	**Total** **(*N* = 313)**	**CHA2DS2-VASc score** **(*****N*** **= 313)**		***P-*value**
		** <6 (*N* = 220)**	**≥6 (*N* = 93)**	
Age, yrs	69.8 ± 11.2	67.7 ± 11.3	74.6 ± 9.2	<0.001
Male sex	128 (40.9)	90 (34.5)	38 (55.9)	<0.001
BMI (kg/m^2^)	24.9 ± 4.8	24.7 ± 3.99	25.2 ± 6.3	0.410
**Medical history**				
Hypertension	274 (87.5)	185 (84.1)	89 (95.7)	0.004
Diabetes mellitus	179 (57.2)	105 (47.7)	74 (79.6)	<0.001
Hyperlipidemia	173 (55.3)	111 (50.5)	62 (66.7)	0.008
Congestive heart failure	164 (52.4)	96 (43.6)	68 (73.1)	<0.001
Prior stroke or TIA	102 (32.6)	35 (15.9)	67 (72.0)	<0.001
Prior MI	85 (27.2)	56 (25.5)	29 (31.2)	0.300
Prior PCI	80 (25.6)	48 (21.8)	32 (34.4)	0.020
Prior CABG	17 (5.4)	10 (4.5)	7 (7.5)	0.290
Peripheral artery disease	57 (18.2)	29 (13.2)	28 (30.1)	<0.001
Stage of CKD				0.690
Stage 3	136 (43.5)	93 (42.6)	43 (46.2)	
Stage 4	47 (15.0)	32 (14.6)	15 (16.1)	
Stage 5	129 (41.2)	94 (42.9)	43 (46.2)	
Clinical presentation				0.820
STEMI	78 (24.9)	57 (25.9)	21 (22.6)	
NSTEMI	112 (35.8)	78 (35.5)	34 (36.6)	
UA	123 (39.3)	85 (38.6)	38 (40.9)	
**Laboratory measures**				
Hemoglobin, g/dl	112.6 ± 121.6	114.8 ± 21.8	107.2 ± 20.5	0.004
Platelet count, × 10^9^/l	194 ± 61	194.3 ± 59.7	189.8 ± 63.1	0.520
LDL, mmol/l	2.5 ± 0.9	2.59 ± 0.88	2.33 ± 0.99	0.005
Serum creatinine, mg/dl	4.33 ± 3.73	4.61 ± 3.94	3.66 ± 3.11	0.110
Uric acid	408 ± 134	404 ± 133	419 ± 136	0.380
Homocysteine, μmol/l	23.6 ± 26.3	24.0 ± 21.4	22.7 ± 35.5	0.007
D-dimer, mg/l	1.30 ± 1.78	1.28 ± 1.93	1.33 ± 1.38	0.060
LVEF	55 ± 12	55 ± 11	55 ± 13	0.450
Reperfusion therapy	212 (67.7)	156 (70.9)	56 (60.2)	0.060
**Medical therapy at admission**				
Aspirin	301 (96.2)	211 (95.9)	90 (96.8)	0.720
P2Y12 receptor antagonist	297 (94.9)	208 (94.5)	89 (95.7)	0.670
Stain	303 (96.8)	213 (96.8)	90 (96.8)	0.980
β-blockers	286 (91.4)	201 (91.4)	85 (91.4)	0.990

*ACEI, angiotensin-converting enzyme inhibitors; ARB, angiotensin receptor blockers; BMI, body mass index; CKD, chronic kidney disease; CABG, coronary artery bypass grafting; LDL, Low-density lipoprotein; LVEF, left ventricular ejection fraction; MI, myocardial infarction; NSTEMI, non-ST-segment elevation myocardial infarction; STEMI, ST-segment elevation myocardial infarction; TIA, transient ischemic attacks; UA, unstable angina*.

**Figure 1:**
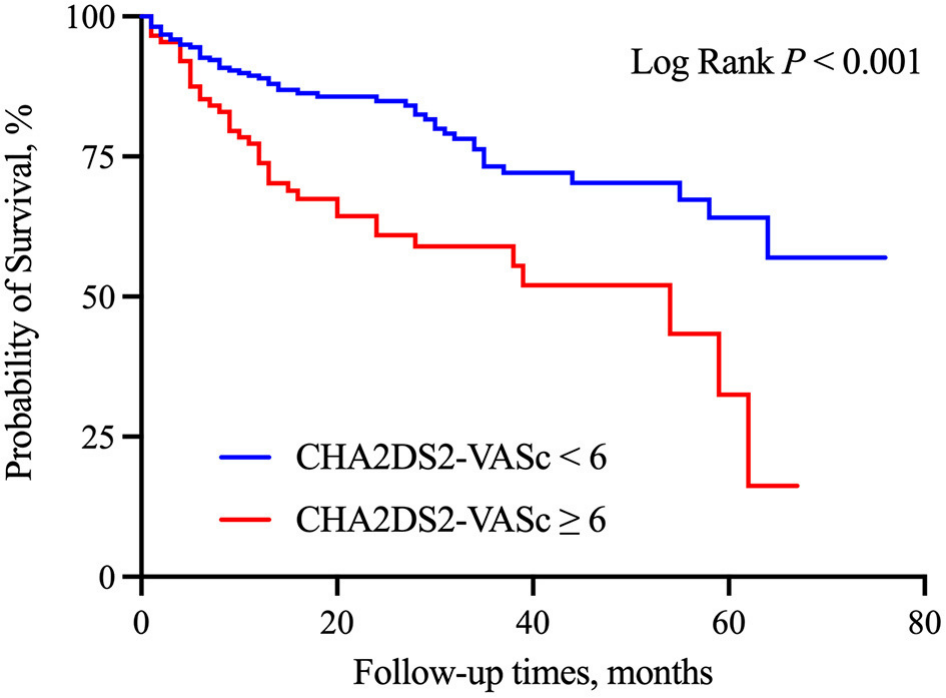
Kaplan-Meier survival curves for all-cause mortality by CHA_2_DS_2_-VASc score.

**Figure 2:**
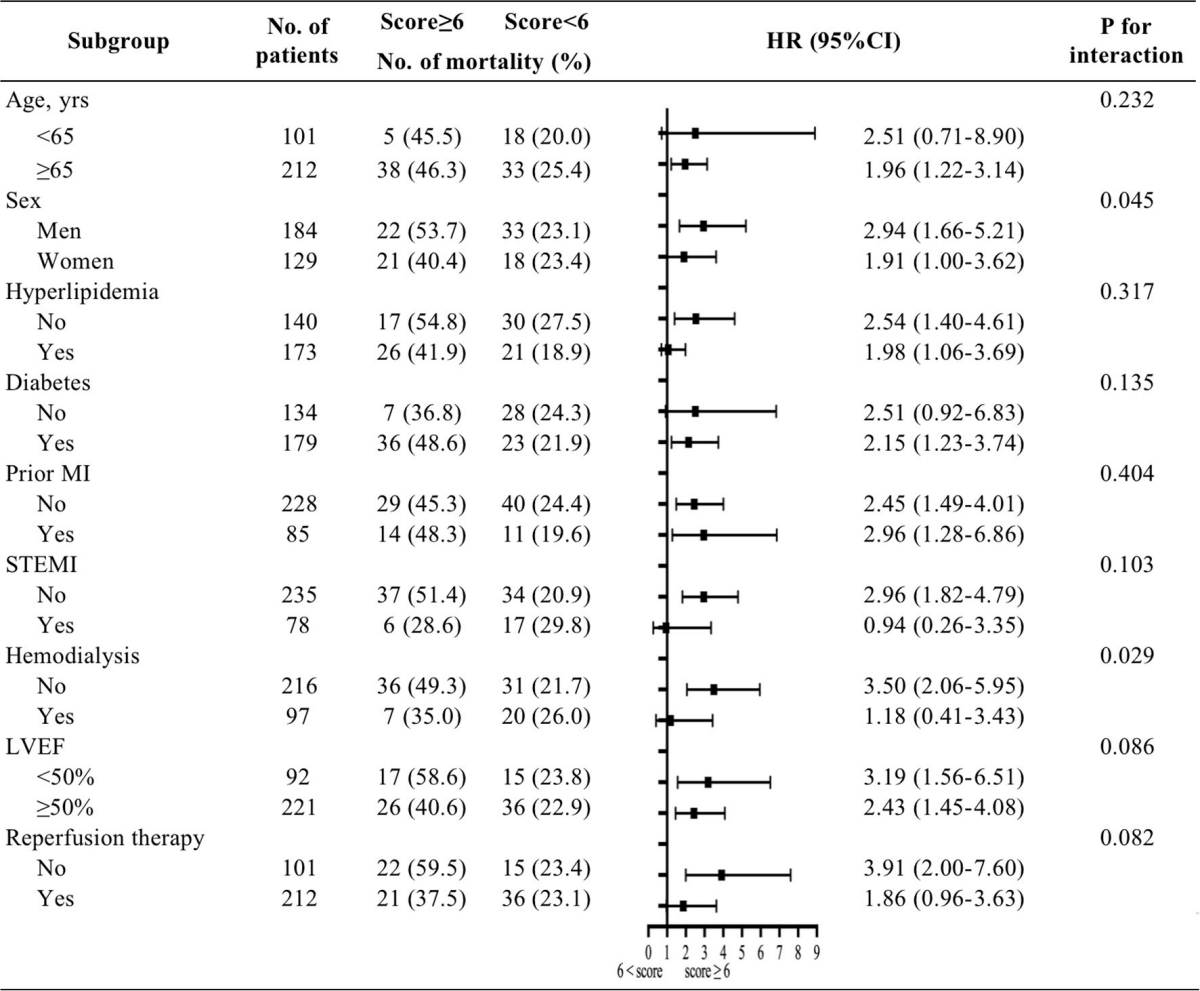
Predictive value of CHA_2_DS_2_-VASC score for all-cause mortality in different subgroups.

The median follow-up time was 23.0 months (interquartile range: 12–38 months). During the follow-up period, a total of 94 patients (30.0%) died. High CHA_2_DS_2_-VASC scores were associated with a higher risk of mortality (46.2 vs. 23.2%, *p* < 0.001). Kaplan-Meier curves for patients who were stratified by CHA_2_DS_2_-VASC scores are presented in Figure 1. The cumulative incidence of all-cause mortality (Log-rank test, *p* < 0.001) was higher in the high CHA_2_DS_2_-VASC score group than in the low CHA_2_DS_2_-VASC score group. We performed Cox univariate and multivariate analyses using the low CHA_2_DS_2_-VASc score group as the reference group. The HR for all-cause mortality was 2.49 (95% CI: 1.66–3.74, *p* < 0.001). After adjusting for hypertension, diabetes, prior myocardial infarction, and CKD stage, the HR of all-cause mortality was 2.029 (95% CI: 1.33–3.10, *p* = 0.001). The HR of all-cause mortality was largely unchanged after adding all other variables with *p* < 0.10 in the univariate analysis (HR: 2.027, 95% CI: 1.26–3.27, *p* < 0.001). The univariate analysis of factors that were related to all-cause mortality is presented in Table 2. The multivariate analyses between the CHA_2_DS_2_-VASc score group and outcomes are shown in Table 3. A significant between-group difference in outcome was found in the subgroup analyses of sex [HR: 2.94 (95% CI: 1.66–5.21) for men; HR: 1.91 (95% CI: 1.00–3.61) for women; *p* = 0.045]. A similar result was found for death in the subgroup analyses of hemodialysis. No significant interactions were found between the other subgroups and CHA_2_DS_2_-VASC scores for the prediction of all-cause mortality. The results of the subgroup analyses are shown in Figure 2.

**Table 2:** Cox regression analysis of factors related to all-cause mortality.

**Parameter**	**Univariate (all-cause mortality)**	
	**HR (95% CI)**	***P-*value**
Age^a^	1.02 (1.00–1.04)	0.024
Male sex (vs. Female)	0.98 (0.65–1.47)	0.920
BMI^b^	0.79(0.53–1.19)	0.260
Prior PCI	1.13 (0.72–1.78)	0.590
Prior CABG	1.38 (0.60–3.16)	0.470
**Stage of CKD**		
Stage 3	1 (ref)	..
Stage 4	1.72 (1.11–2.67)	0.015
Stage 5	2.12 (1.14–4.31)	0.019
**Diagnosis**		
UA	1 (ref)	..
NSTEMI	1.30 (0.76–2.25)	0.340
STEMI	1.80 (1.12–2.89)	0.015
Hemoglobin, g/dl	0.990 (0.981–0.999)	0.027
Platelet count, × 10^9^/l	0.998 (0.994–1.000)	0.180
LDL, mmol/l	0.87 (0.69–1.10)	0.250
Uric acid, μmol/l	0.999 (0.998–1.001)	0.360
Serum creatinine, mg/dl	1.04 (0.98–1.09)	0.180
Homocysteine, μmol/l	1.004 (0.999–1.010)	0.100
D-dimer, mg/l	1.08 (1.00–1.164)	0.041
LVEF (≥50 vs. <50)	0.74 (0.48–1.13)	0.160
Reperfusion therapy	0.68 (0.45–1.04)	0.070
Aspirin	1.24 (0.39–3.93)	0.710
P2Y12 receptor antagonist	1.55 (0.49–4.88)	0.460
Stain	1.58 (0.38–6.43)	0.520
β-blockers	1.37 (0.60–3.15)	0.450
ACEI/ARB	1.58 (0.39–6.43)	0.520
CHA2DS2-VASc score^c^ ≥ 6	2.48 (1.65–3.73)	<0.001

a *Per 1 unit increase*.

b *BMI ≥ 24 kg/m^2^ vs. BMI < 24 kg/m^2^*.

c *CHA2DS2-VASc score ≥ 6 score vs. CHA2DS2-VASc score < 6 score*.

*ACEI, angiotensin-converting enzyme inhibitors; ARB, angiotensin receptor blockers; BMI, body mass index; CKD, chronic kidney disease; CABG, coronary artery bypass grafting; LDL, Low-density lipoprotein; LVEF, left ventricular ejection fraction; MI, myocardial infarction; NSTEMI, non-ST-segment elevation myocardial infarction; STEMI, ST-segment elevation myocardial infarction; TIA, transient ischemic attacks; UA, unstable angina*.

**Table 3:** Adjusted hazard ratios of all-cause mortality by high CHA2DS2-VASc score (≥6 points) relative to low CHA2DS2-VASc score (<6 points).

**Model adjustment**	**All-cause mortality**	
	**HR (95% CI)**	***P-*value**
Unadjusted	2.49 (1.66–3.74)	< 0.001
Model 1	2.49 (1.66–3.74)	< 0.001
Model 2	2.029 (1.33–3.10)	0.001
Model 3	2.027 (1.26–3.27)	0.004

*Model 1, adjusted for age, sex; Model 2, adjusted for age, sex, hypertension, diabetes, prior MI, stage of CKD; Model 3, adjusted for age, sex, hypertension, diabetes, Hyperlipidemia, prior MI, stage of CKD, diagnosis, Cr, LVEF, reperfusion therapy and medication therapy at admission*.

*HR, hazard ratios; CI, confidence interval*.

## Discussion

The present study found that CHA_2_DS_2_-VASC scores were associated with worse clinical outcome in CKD patients with ACS. High scores (≥6) were an independent predictor of all-cause mortality and may be useful for risk stratification. The subgroup analyses indicated that high scores were a slightly better predictor of all-cause mortality in men than in women and in patients who did not undergo hemodialysis. Compared with patients with low scores, patients with high scores were more often older and women and had a higher prevalence of comorbidities. Additionally, patients with high scores were less likely to receive reperfusion therapy in clinical practice.

Acute coronary syndrome is a common critical cardiovascular disease and primary focus of cardiologists. Benefiting from the application of stents, the mortality rate of ACS has gradually decreased over the past decade (15). Patients with coronary disease and CKD, especially end-stage kidney disease, have a very high risk of cardiovascular events (16, 17). The high rate of all-cause mortality in the present study aligns with the high-risk feature of these patients in previous studies. Despite having worse outcomes after a cardiovascular event, patients with CKD are often excluded from the majority of ACS or heart failure cardiovascular outcome trials (18). The reasons for this are likely multifactorial, such as the potential for diminished effects of medical treatment and coronary intervention in trials, complex pathophysiological mechanisms that contribute to cardiovascular disease, safety concerns, and trial recruitment difficulties (19). Therefore, clinical evidence from the general population may not be suitable for this specific patient population. The Framingham risk score is the most well-validated coronary artery disease risk prediction tool, but it has been shown to have poor overall accuracy in predicting cardiac events in individuals with CKD (20). Data from GRACE indicated that the GRACE risk score underestimates the risk of major events in end-stage kidney disease patients who undergo dialysis (21). Moreover, the inclusion of multiple types of variables and relatively complex calculation significantly limit clinical utility of the GRACE risk score (8).

The CHA_2_DS_2_-VASC score is a validated and extensively used score to estimate thromboembolic risk in patients with atrial fibrillation, consisting of several cardiovascular risk factors (9). Among these factors, old age, hypertension, diabetes, and heart failure have been proven to influence the prognosis of cardiovascular disease (4, 5, 22–24). Prior stroke is also associated with a high risk of major adverse cardiovascular and cerebrovascular events (25). Sex differences in the epidemiology, manifestation, pathophysiology, and outcome of cardiovascular disease have been observed in previous studies (26). Therefore, all components of the CHA_2_DS_2_-VASc score have a close association with the prognosis of cardiovascular disease. Tufan Cinar et al. evaluated 267 patients with mechanical mitral valve thrombosis and found that a CHA_2_DS_2_-VASc score ≥ 2.5 was associated with a higher risk of prosthetic valve thrombosis (27). Several recent studies evaluated the predictive value of the CHA_2_DS_2_-VASc score for clinical outcome. A large real-world cohort study reported that CHA_2_DS_2_-VASc scores were significantly associated with mortality in heart failure patients (11). Hsu et al. reported the predictive value of CHA_2_DS_2_-VASc scores for all-cause mortality and cardiovascular mortality in CKD patients without ACS (28). A similar study found that CHA_2_DS_2_-VASc scores were strongly associated with 1-year mortality and cardiovascular risk in hemodialysis patients (29). Studies that investigated patients with ST-elevation myocardial infarction showed that CHA_2_DS_2_-VASc scores were an independent predictor of no-reflow and an independent predictor of in-hospital and long-term mortality in patients who underwent primary percutaneous coronary intervention (30–32). Although the association between CHA_2_DS_2_-VASc score and clinical outcome in ACS patients without CKD or CKD patients without ACS have been estimated, the value of these scores in ACS patients with CKD is unclear. In the present study, we found a significant association between CHA_2_DS_2_-VASc scores and all-cause mortality in ACS patients with CKD, which may be useful for the risk stratification of these patients. The mean CHA_2_DS_2_-VASc score in the present study was significantly higher than in patients without CKD in a previous study, which may help explain the high mortality in ACS patients with CKD. Variables that are included in the CHA_2_DS_2_-VASC score can be readily found in patients' medical histories. Furthermore, CHA_2_DS_2_-VASC scores may be useful for quickly identifying very high-risk ACS patients with CKD.

The present study has limitations. This was a single-center, retrospective study. We were unable to control the variables that were included in the analyses given the study's observational design. In addition to traditional cardiovascular risk factors (e.g., diabetes and hypertension), non-traditional CKD-related CVD risk factors (e.g., mineral and bone disease abnormalities, vascular calcification, inflammation, and oxidative stress) may also play an important role in the prognosis of cardiovascular disease (4). However, we focused on the prognostic value of the CHA_2_DS_2_-VASc scoring system in ACS patients with CKD, based on variables that were readily obtained from the patients' medical records. Another limitation was that the sample size was not sufficiently large to evaluate prognostic value in dialysis and non-dialysis populations separately. Future studies should integrate CHA_2_DS_2_-VASc scores with non-traditional CKD-related CVD risk factors and develop and validate novel CVD risk prediction scores for the CKD population and dialysis population.

In conclusion, CHA_2_DS_2_-VASc scores were an independent predictive factor for mortality in ACS patients with CKD. The CHA_2_DS_2_-VASc scoring system is a simple and practical method for identifying very high-risk ACS patients among the CKD population. Further studies are needed to evaluate whether CHA_2_DS_2_-VASc scoring can improve the management and outcome of this high-risk population.

## Data Availability Statement

The raw data supporting the conclusions of this article will be made available by the authors, without undue reservation.

## Ethics Statement

The studies involving human participants were reviewed and approved by the Research Ethical Review Committee of China-Japan Friendship Hospital. Written informed consent for participation was not required for this study in accordance with the national legislation and the institutional requirements.

## Author Contributions

YW, JR, and JZ: study design and manuscript preparation. YG, QL, CW, EX, YT, ZG, ZY, PL, YL, and XY: data collection. YW and YG: data analysis and interpretation. All authors contributed to the article and approved the submitted version.

## Funding

This work was supported by the National Natural Science Foundation of China (No. 91639110), National Key Clinical Specialty Construction Project (No. 2020-QTL-009), Natural Science Foundation of Beijing Municipality (No. 7172195), and Science Foundation of China-Japan Friendship Hospital (No. 2020-HX-40).

## Conflict of Interest

The authors declare that the research was conducted in the absence of any commercial or financial relationships that could be construed as a potential conflict of interest.

## Publisher's Note

All claims expressed in this article are solely those of the authors and do not necessarily represent those of their affiliated organizations, or those of the publisher, the editors and the reviewers. Any product that may be evaluated in this article, or claim that may be made by its manufacturer, is not guaranteed or endorsed by the publisher.

## References

[1] Bikbov B Purcell CA Levey AS Smith M Abdoli A Abebe M. Global, regional, and national burden of chronic kidney disease, 1990–2017: a systematic analysis for the Global Burden of Disease Study 2017. Lancet. (2020) 395:709–33. 10.1016/S0140-6736(20)30045-3 32061315 PMC7049905

[2] Zhang L Zhao MH Zuo L Wang Y Yu F Zhang H. China kidney disease network (CK-NET) 2016 annual data report. Kidney Int Suppl 2011. (2020) 10:e97–e185. 10.1016/j.kisu.2020.09.001 33304640 PMC7716083

[3] US Renal Data System 2019. Annual data report: epidemiology of kidney disease in the United States. Am J Kidney Dis. (2020) 75(1 Suppl. 1):A6–A7. 10.1053/j.ajkd.2019.09.003. 31704083

[4] Sarnak MJ Amann K Bangalore S Cavalcante JL Charytan DM Craig JC. Chronic kidney disease and coronary artery disease: JACC state-of-the-art review. J Am Coll Cardiol. (2019) 74:1823–38. 10.1016/j.jacc.2019.08.1017 31582143

[5] Jankowski J Floege J Fliser D Bohm M Marx N. Cardiovascular disease in chronic kidney disease: pathophysiological insights and therapeutic options. Circulation. (2021) 143:1157–72. 10.1161/CIRCULATIONAHA.120.050686 33720773 PMC7969169

[6] Collet JP Thiele H Barbato E Barthelemy O Bauersachs J Bhatt DL. 2020 ESC Guidelines for the management of acute coronary syndromes in patients presenting without persistent ST-segment elevation. Eur Heart J. (2021) 42:1289–367. 10.1093/eurheartj/ehab088 32860058

[7] Ibanez B James S Agewall S Antunes MJ Bucciarelli-Ducci C Bueno H. 2017 ESC Guidelines for the management of acute myocardial infarction in patients presenting with ST-segment elevation: the task force for the management of acute myocardial infarction in patients presenting with ST-segment elevation of the European Society of Cardiology (ESC). Eur Heart J. (2018) 39:119–77. 10.1093/eurheartj/ehx393 28886621

[8] Fox KA Dabbous OH Goldberg RJ Pieper KS Eagle KA Van de Werf F. Prediction of risk of death and myocardial infarction in the six months after presentation with acute coronary syndrome: prospective multinational observational study (GRACE). BMJ. (2006) 333:1091. 10.1136/bmj.38985.646481.55 17032691 PMC1661748

[9] Lip GYH Nieuwlaat R Pisters R Lane DA Crijns HJGM. Refining clinical risk stratification for predicting stroke and thromboembolism in atrial fibrillation using a novel risk factor-based approach: the euro heart survey on atrial fibrillation. Chest. (2010) 137:263–72. 10.1378/chest.09-1584 19762550

[10] Hindricks G Potpara T Dagres N Arbelo E Bax JJ Blomstrom-Lundqvist C. 2020 ESC Guidelines for the diagnosis and management of atrial fibrillation developed in collaboration with the European Association for Cardio-Thoracic Surgery (EACTS): the task force for the diagnosis and management of atrial fibrillation of the European Society of Cardiology (ESC) Developed with the special contribution of the European Heart Rhythm Association (EHRA) of the ESC. Eur Heart J. (2021) 42:373–498. 10.1093/eurheartj/ehab648 32860505

[11] Shuvy M Zwas DR Keren A Gotsman I. Value of the CHA DS -VASc score for predicting outcome in patients with heart failure. ESC Heart Fail. (2020) 7:2553–60. 10.1002/ehf2.12831 32614479 PMC7524134

[12] Renda G Ricci F Patti G Aung N Petersen SE Gallina S. CHADSVASc score and adverse outcomes in middle-aged individuals without atrial fibrillation. Eur J Prev Cardiol. (2019) 26:1987–97. 10.1177/2047487319868320 31409109

[13] Sanchez Fernandez JJ Ortiz MR Ballesteros FM Luque CO Penas ER Ortega MD. CHADS-VASc score as predictor of stroke and all-cause death in stable ischaemic heart disease patients without atrial fibrillation. J Neurol. (2020) 267:3061–8. 10.1007/s00415-020-09961-7 32529579

[14] Di Lullo L House A Gorini A Santoboni A Russo D Ronco C. Chronic kidney disease and cardiovascular complications. Heart Fail Rev. (2015) 20:259–72. 10.1007/s10741-014-9460-9 25344016

[15] Widimsky P Crea F Binder RK Lüscher TF. The year in cardiology 2018: acute coronary syndromes. Eur Heart J. (2019) 40:271–82. 10.1093/eurheartj/ehy904 30601993

[16] Go AS Chertow GM Fan D McCulloch CE Hsu C-y. Chronic kidney disease and the risks of death, cardiovascular events, and hospitalization. New Engl J Med. (2004) 351:1296–305. 10.1056/NEJMoa041031 15385656

[17] Matsushita K van der Velde M Astor BC Woodward M Levey AS de Jong PE. Association of estimated glomerular filtration rate and albuminuria with all-cause and cardiovascular mortality in general population cohorts: a collaborative meta-analysis. Lancet. (2010) 375:2073–81. 10.1016/S0140-6736(10)60674-5 20483451 PMC3993088

[18] Konstantinidis I Nadkarni GN Yacoub R Saha A Simoes P Parikh CR. Representation of patients with kidney disease in trials of cardiovascular interventions: an updated systematic review. JAMA Intern Med. (2016) 176:121–4. 10.1001/jamainternmed.2015.6102 26619332

[19] Zannad F Rossignol P. Cardiovascular outcome trials in patients with advanced kidney disease: time for action. Circulation. (2017) 135:1769–71. 10.1161/CIRCULATIONAHA.117.027338 28483826

[20] Weiner DE Tighiouart H Elsayed EF Griffith JL Salem DN Levey AS. The Framingham predictive instrument in chronic kidney disease. J Am Coll Cardiol. (2007) 50:217–24. 10.1016/j.jacc.2007.03.037 17631213

[21] Gurm HS Gore JM Anderson FA Wyman A Fox KAA Steg PG. Comparison of acute coronary syndrome in patients receiving versus not receiving chronic dialysis (from the Global Registry of Acute Coronary Events [GRACE] Registry). Am J Cardiol. (2012) 109:19–25. 10.1016/j.amjcard.2011.07.062 21974963

[22] Flint AC Conell C Ren X Banki NM Chan SL Rao VA. Effect of systolic and diastolic blood pressure on cardiovascular outcomes. N Engl J Med. (2019) 381:243–51. 10.1056/NEJMoa1803180 31314968

[23] North BJ Sinclair DA. The intersection between aging and cardiovascular disease. Circ Res. (2012) 110:1097–108. 10.1161/CIRCRESAHA.111.246876 22499900 PMC3366686

[24] Simons LA Simons J. Diabetes and coronary heart disease. N Engl J Med. (1998) 339:2314. 10.1056/NEJM199812033392314 9867543

[25] Wang H Ning X Zhu C Yin D Feng L Xu B. Prognostic significance of prior ischemic stroke in patients with coronary artery disease undergoing percutaneous coronary intervention. Catheteriz Cardiovasc Interv. (2019) 93:787–92. 10.1002/ccd.28057 30618111

[26] Regitz-Zagrosek V Kararigas G. Mechanistic pathways of sex differences in cardiovascular disease. Physiol Rev. (2017) 97:1–37. 10.1152/physrev.00021.2015 27807199

[27] Cinar T Hayiroglu MI Tanik VO Arugaslan E Keskin M Uluganyan M. The predictive value of the CHA2DS2-VASc score in patients with mechanical mitral valve thrombosis. J Thromb Thrombolysis. (2018) 45:571–7. 10.1007/s11239-018-1640-3 29524112

[28] Vodošek Hojs N Ekart R Bevc S Piko N Hojs R. CHA2DS2-VASc score as a predictor of cardiovascular and all-cause mortality in chronic kidney disease patients. Am J Nephrol. (2021) 52:404–11. 10.1159/000516121 33975308

[29] Schamroth Pravda M Cohen Hagai K Topaz G Schamroth Pravda N Makhoul N Shuvy M. Assessment of the CHA2DS2-VASc score in predicting mortality and adverse cardiovascular outcomes of patients on hemodialysis. Am J Nephrol. (2020) 51:635–40. 10.1159/000508836 32702703

[30] Kim KH Kim W Hwang SH Kang WY Cho SC Kim W. The CHA2DS2VASc score can be used to stratify the prognosis of acute myocardial infarction patients irrespective of presence of atrial fibrillation. J Cardiol. (2015) 65:121–7. 10.1016/j.jjcc.2014.04.011 24972564

[31] Ipek G Onuk T Karatas MB Gungor B Osken A Keskin M. CHA2DS2-VASc score is a predictor of no-reflow in patients with ST-segment elevation myocardial infarction who underwent primary percutaneous intervention. Angiology. (2016) 67:840–5. 10.1177/0003319715622844 26685178

[32] Bozbay M Uyarel H Cicek G Oz A Keskin M Murat A. CHA2DS2-VASc score predicts in-hospital and long-term clinical outcomes in patients with st-segment elevation myocardial infarction who were undergoing primary percutaneous coronary intervention. Clin Appl Thromb Hemost. (2017) 23:132–8. 10.1177/1076029616646874 27170782

