# Supplementary material for: Predictive Value of the CHA2DS2-VASc Score for Mortality in Hospitalized Acute Coronary Syndrome Patients With Chronic Kidney Disease
Source: Front Cardiovasc Med. 2022 Mar 16;9:790193. doi: 10.3389/fcvm.2022.790193 (PMC8965867; doi:10.3389/fcvm.2022.790193)
Supplement: Supplementary file 1 [file Data_Sheet_1.pdf]

Supplementary material

Receiver operating characteristic (ROC) analysis which showed that the best cut-off value of the CHA2DS2-VASc score to predict mortality

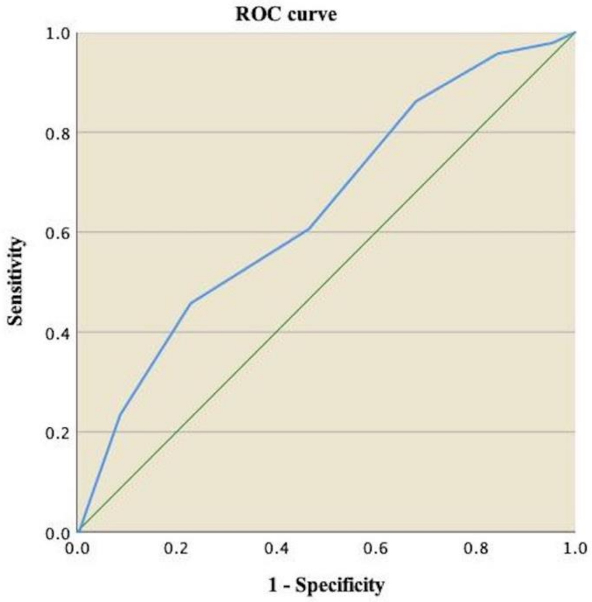

CHA2DS2-VASc score	Sensitivity	1 - Specificity	Youden's J statistic
0	1	1	
2	0.979	0.954	1.025
3	0.957	0.845	1.112
4	0.862	0.68	1.182
5	0.606	0.466	1.14
6	0.457	0.228	1.229
7	0.234	0.087	1.147
8	0.053	0.023	1.03
9	0	0.005	0.995
10	0	0	1

We performed a receiver operating characteristic analysis that showed that the best cut-off value of the CHA_2DS_2-VASc score to predict mortality was ≥ 6 with 45.7% sensitivity and 77.2% specificity (area under curve: 0.64; 95% confidence interval [CI]: 0.58-0.71, $p < 0.001$).
